# Effect of Spherical Elements of Biosensors and Bioreactors on the Physicochemical Properties of a Peroxidase Protein

**DOI:** 10.3390/polym13101601

**Published:** 2021-05-15

**Authors:** Yuri D. Ivanov, Vadim Yu. Tatur, Tatyana O. Pleshakova, Ivan D. Shumov, Andrey F. Kozlov, Anastasia A. Valueva, Irina A. Ivanova, Maria O. Ershova, Nina D. Ivanova, Victor V. Repnikov, Igor N. Stepanov, Vadim S. Ziborov

**Affiliations:** 1 Institute of Biomedical Chemistry, 119121 Moscow, Russia; t.pleshakova1@gmail.com (T.O.P.); shum230988@mail.ru (I.D.S.); afkozlow@mail.ru (A.F.K.); varuevavarueva@gmail.com (A.A.V.); i.a.ivanova@bk.ru (I.A.I.); motya00121997@mail.ru (M.O.E.); ziborov.vs@yandex.ru (V.S.Z.); 2 Joint Institute for High Temperatures of the Russian Academy of Sciences, 125412 Moscow, Russia; 3 Foundation of Perspective Technologies and Novations, 115682 Moscow, Russia; v_tatur@mail.ru (V.Y.T.); ninaivan1972@gmail.com (N.D.I.); fptn@mail.ru (I.N.S.); 4 Skryabin Moscow State Academy of Veterinary Medicine and Biotechnology, 109472 Moscow, Russia; 5 Bruker Ltd., 119017 Moscow, Russia; viktor.repnikov@bruker.com

**Keywords:** atomic force microscopy, horseradish peroxidase, protein aggregation, electromagnetic field, bioreactor

## Abstract

External electromagnetic fields are known to be able to concentrate inside the construction elements of biosensors and bioreactors owing to reflection from their surface. This can lead to changes in the structure of biopolymers (such as proteins), incubated inside these elements, thus influencing their functional properties. Our present study concerned the revelation of the effect of spherical elements, commonly employed in biosensors and bioreactors, on the physicochemical properties of proteins with the example of the horseradish peroxidase (HRP) enzyme. In our experiments, a solution of HRP was incubated within a 30 cm-diameter titanium half-sphere, which was used as a model construction element. Atomic force microscopy (AFM) was employed for the single-molecule visualization of the HRP macromolecules, adsorbed from the test solution onto mica substrates in order to find out whether the incubation of the test HRP solution within the half-sphere influenced the HRP aggregation state. Attenuated total reflection Fourier transform infrared spectroscopy (ATR-FTIR) was employed in order to reveal whether the incubation of HRP solution within the half-sphere led to any changes in its secondary structure. In parallel, spectrophotometry-based estimation of the HRP enzymatic activity was performed in order to find out if the HRP active site was affected by the electromagnetic field under the conditions of our experiments. We revealed an increased aggregation of HRP after the incubation of its solution within the half-sphere in comparison with the control sample incubated far outside the half-sphere. ATR-FTIR allowed us to reveal alterations in HRP’s secondary structure. Such changes in the protein structure did not affect its active site, as was confirmed by spectrophotometry. The effect of spherical elements on a protein solution should be taken into account in the development of the optimized design of biosensors and bioreactors, intended for performing processes involving proteins in biomedicine and biotechnology, including highly sensitive biosensors intended for the diagnosis of socially significant diseases in humans (including oncology, cardiovascular diseases, etc.) at early stages.

## 1. Introduction

Proteins, representing polymers of amino acids, are among the main types of biopolymers, playing various vital functions in living organisms [1]. Proteins can function as single-macromolecule structures or in the form of various complexes, including (but not limited to) protein oligomers and protein-protein, protein–nucleic acid, and protein–small molecule complexes [1]. In modern life, electromagnetic fields are widely employed, influencing living organisms. The functionality of protein systems (including enzymatic ones) can be altered under the action of magnetic [2] and electromagnetic fields [3,4,5,6,7,8,9,10]. In this way, in previous studies, we demonstrated that electric fields, triboelectrically induced by liquid flow through polymeric pipes of thermal stabilization coils, influence the adsorbability of the horseradish peroxidase (HRP) enzyme protein onto mica substrates [5,6,7]. Moreover, under certain conditions, its enzymatic activity can also be affected by the flow-induced field [6]. A 40 min exposure to an external ultra-weak (10^−12^ W/cm^2^) 2.3 GHz knotted electromagnetic field was also shown to have an effect on the HRP aggregation state upon its adsorption onto mica [4]. Lopes et al. [9] found that 2450 MHz [11] microwave radiation can cause a significant (up to >80%) loss in the HRP enzymatic activity after a 0.5 h treatment at 60 °C and 60 W microwave power. Hamedi et al. demonstrated partial unfolding of adult hemoglobin (HbA) after exposure to a 940 MHz circularly polarized electromagnetic field [8]. As regards HRP, a 52 mT static magnetic field was also shown to impact its enzymatic activity and optimum pH by inducing changes in its structure [2].

As regards proteins, alterations in their functionality can manifest themselves not only as direct changes in functional activity [2,3,9], but also in the form of changes in adsorbability [5,6,7,10] onto functional surfaces of biosensors and bioreactors. The latter is of importance for biotechnology applications, where bioreactors with surface-immobilized enzymes are widely employed [12,13].

Spherical elements are commonly used in the construction of bioreactors, including bioreactor bottoms [14,15]. Previous theoretical studies demonstrated the ability of large pyramidal objects to concentrate weak electromagnetic radiation near their surface and within their volume, which is attributed to resonance phenomena [16]. These phenomena are supposed to be the very cause of the effect of incubation near a pyramidal structure on HRP adsorbability from aqueous solutions onto mica [10]. In this connection, it should be noted that electromagnetic field-induced protein aggregation can lead to the occurrence of pathologies in the body, for instance by influencing the rheological properties of blood [17,18]. It should be emphasized that the effects on blood rheology were observed in the case of electromagnetic fields of commercial 50 Hz mains frequency [17], commonly employed in both industry and everyday life. Since reaction vessels with spherical bottoms are widely employed in industry, it is particularly important to study the influence of external electromagnetic fields of commercial frequency, concentrated by spherical construction elements of bioreactors, on protein systems.

The horseradish peroxidase (HRP) enzyme protein is widely employed in biotechnology [13]. The wide use in both research and industrial applications makes it important to study the influence of electromagnetic fields of commercial frequency on its properties. Moreover, the availability of detailed and comprehensive information about its structure and physicochemical properties makes it easier to interpret the effects observed in the experiments, and this is why HRP is a useful model object in studying the external impacts on the properties of proteins. Structurally, HRP represents a 40–44 kDa [19,20] heme-containing enzyme glycoprotein [21], which contains 18–27% structure-stabilizing carbohydrate residues [20,22]. In micromolar aqueous solutions, HRP is prone to aggregation [23], while being presented in monomeric form at ultra-low concentrations [24].

Atomic force microscopy (AFM) represents a high-resolution method commonly employed in studying single-polymer macromolecules at the nanoscale [25]. Studying biopolymers such as proteins [24,26,27,28,29] and nucleic acids [30,31,32,33,34] is one of the main directions in the development of AFM applications [35]. Owing to its extremely high (~0.1 nm) height resolution, AFM allows one to visualize single biological macromolecules and their complexes. Moreover, AFM allows one to determine the physicochemical properties of proteins at the single-molecule level, such as Young’s modulus [29], aggregation state [4,27], and enzymatic activity [29,36]. Since AFM allows one to study single-polymer macromolecules, operating at the nanoscale, it allows one to reveal subtle effects [37], which are indistinguishable by macroscopic methods [4]. In our previous studies, the AFM-based approach allowed us to reveal the effects of external electromagnetic fields on the adsorption properties and aggregation state of HRP [4,5,6,7,10].

Our present study concerned the atomic force microscopy (AFM)-based revelation of the effect of a half-spherical element, whose shape was similar to the commonly used construction elements of bioreactor vessels, on the properties of the HRP model enzyme protein. The study was performed under common laboratory conditions, and the experimental setup, including the spherical element, was devoid of any electromagnetic shielding. We emphasize that in our experiments reported herein, external electromagnetic radiation was not generated intentionally by using specialized generators, as opposed to our previously reported experiments [4]. In addition, the attenuated total reflection Fourier transform infrared spectroscopy (ATR-FTIR) method was employed in order to reveal whether or not the incubation of the HRP solution within the half-sphere led to any changes in its secondary structure. Moreover, spectrophotometry-based estimation of the HRP’s enzymatic activity was performed in order to find out whether or not the HRP active site was affected by the electromagnetic field under the conditions of our experiments. In our experiments reported herein, an increased aggregation of HRP after the incubation of its aqueous solution inside a titanium half-sphere was observed. The ATR-FTIR data obtained indicated alterations in HRP’s secondary structure after the incubation of its solution in the center of the half-sphere. At the same time, spectrophotometry results indicated no change in HRP’s enzymatic activity. These results indicated the importance of further studying how electromagnetic fields of commercial (50 Hz) frequency, generated by various equipment in both industry and everyday life, can affect living systems. This is required in order to develop safety standards regulating the application of electromagnetic field-inducing equipment in industry, where technological vessels with spherical construction elements are commonly employed. This is why the development of novel highly sensitive biosensor systems, which allow one to perform measurements at the single-molecule level, represents a crucial problem in biomedical research. The development of such biosensors will help to better understand the influence of external electromagnetic fields on humans. Moreover, the application of such systems will allow us to solve a number of important problems in biomedicine, including the early diagnosis of somatic and infectious diseases (such as cancer, cardiovascular diseases, hepatitis, and other viral infections) in humans.

## 2. Materials and Methods

### 2.1. Chemicals and Protein

Peroxidase from horseradish (HRP-C; Cat.# P6782) and 2,2′-azino-bis(3-ethylbenzothiazoline-6-sulfonate) (ABTS) were purchased from Sigma (St. Louis, MO, USA). Disodium hydrogen orthophosphate (Na_2_HPO_4_), citric acid, and hydrogen peroxide (H_2_O_2_) were purchased from Reakhim (Moscow, Russia). A 2 mM Dulbecco’s modified phosphate-buffered saline (PBSD buffer) was prepared by dissolving a certain amount of salt mixture (Pierce; Waltham, MA, USA) in deionized ultrapure water. All solutions were prepared using deionized ultrapure water (of 18.2 MΩ×cm resistivity) obtained with a Simplicity UV system (Millipore, Molsheim, France).

### 2.2. Experimental Setup

**Figure 1:**
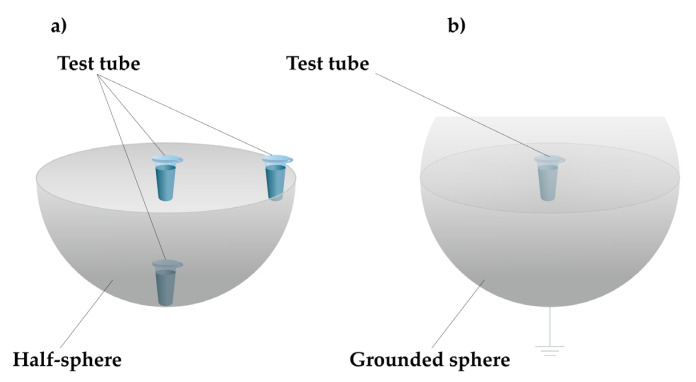
Experimental setup. The test tube containing a 0.1 μM (10^−7^ M) solution of HRP in a 2 mM PBSD buffer was incubated within an ungrounded metallic half-sphere (in its center, near its edge, or at its bottom) (**a**), in the center of a grounded metallic sphere (**b**), or 2 m away from the experimental setup (control experiment).

The experimental setup, employed in the present study, is schematically shown in Figure 1.

In the experimental setup, a 300 mm-diameter, 8 mm-thick titanium half-sphere was employed. For AFM experiments, a 0.1 μM (10^−7^ M) HRP solution was prepared by serial ten-fold dilution of the initial 10^−4^ M solution of the protein with a 2 mM Dulbecco’s modified phosphate-buffered saline (PBSD buffer). A standard 1.7 mL Eppendorf-type polypropylene tube, containing 1 mL of analyzed 0.1 μM solution of HRP in PBSD buffer, was placed within the half-sphere—namely, in its center, near its edge, or at its bottom (as shown in Figure 1a)—and incubated for 40 min. Additionally, in order to determine whether shielding of the protein solution from external electromagnetic fields affected the measurement results, the test solution was incubated in the center of a grounded metallic sphere, as shown in Figure 1b. In control experiments, the sample was incubated 2 m away from the half-sphere.

### 2.3. AFM Sample Preparation

AFM samples were prepared by the direct surface adsorption method [38], similar to [4,5,6,7,10]. Freshly cleaved muscovite mica sheets (SPI, West Chester, PA, USA) were used as the AFM substrates. For AFM sample preparation, each mica sheet was immersed into a separate 1.7 mL Eppendorf-type polypropylene tube containing 800 µL of the analyzed 0.1 μM aqueous HRP solution in a 2 mM PBSD buffer and incubated therein for 10 min at room temperature in a shaker at 600 rpm. After the incubation, each substrate was rinsed with 1 mL of ultrapure water and then dried in air.

### 2.4. AFM Measurements

The surface of mica substrates with HRP molecules, adsorbed from the solutions, which were incubated either within the half-sphere or 2 m away from it, was visualized by AFM. All AFM measurements were performed in tapping mode in air with a Titanium multimode atomic force microscope (NT-MDT, Zelenograd, Russia; the microscope pertains to the equipment of the “Human Proteome” Core Facility of the Institute of Biomedical Chemistry, supported by Ministry of Education and Science of Russian Federation, Agreement 14.621.21.0017, unique project ID: RFMEFI62117X0017) equipped with NSG03 cantilevers (“TipsNano”, Zelenograd, Russia; 47–150 kHz resonant frequency, 0.35–6.1 N/m force constant). The number of frames obtained for each sample was no less than 20. The density of the relative distribution of the imaged objects with height *ρ(h)* was calculated as described elsewhere [39].

Prior to the experiments with the HRP samples, preliminary experiments with use of protein-free 2 mM PBSD buffer instead of protein solution were performed. In these preliminary experiments, no objects with a >0.5 nm height were registered.

AFM operation, obtaining AFM images, their treatment, and exporting the obtained data in ASCII format were performed using the standard NOVA Px software (NT-MDT, Moscow, Zelenograd, Russia) supplied with the atomic force microscope. The number of visualized particles in the obtained AFM images was calculated automatically using a specialized AFM data processing software developed at the Institute of Biomedical Chemistry (Rospatent Registration No. 2010613458).

### 2.5. ATR-FTIR Measurements

In order to reveal possible changes in the HRP secondary structure after the incubation of its solution in the center of the half-sphere, the attenuated total reflection Fourier transform infrared spectroscopy (ATR-FTIR) method was employed. HRP solutions, incubated either in the center of the half-sphere or at a 2 m distance from the half-sphere (control solution), were analyzed with an INVENIO spectrometer (Bruker Scientific LLC, Billerica, MA, USA). The ATR-FTIR measurements were performed in the following way: 12 µL of the analyzed 10^−4^ M HRP solution in 2 mM PBSD buffer were placed into the measuring cell of the spectrometer. Therefore, a high (10^−4^ M) protein concentration was used due to the sensitivity limitations of the spectrometer employed. Each experiment was carried out with three samples, and for each sample, the measurements were performed twice. The number of technical replicate scans in each experiment was 120. Data, obtained in the ATR-FTIR measurements, were presented in standard form provided by the spectrometer operation software. In order to account for the contribution of the PBSD buffer to the resulting spectra, blank measurements with pure protein-free buffer were performed prior to the experiments with the HRP solutions within the same spectral range. The so-obtained ATR-FTIR spectrum of the buffer was subtracted from that of the protein solutions.

### 2.6. Spectrophotometry Measurements

In order to find out whether or not the incubation of the solution of the HRP protein within the half-sphere influenced its enzymatic activity, the latter was estimated by spectrophotometry, employing a technique described in detail by Sanders et al. [40] with ABTS as the reducing substrate, as described in our previously reported studies [4,5,7,10]. Briefly, the rate of change in solution absorbance at 405 nm was measured employing an Agilent 8453 UV-visible spectrophotometer (Agilent Technologies Deutschland GmbH, Waldbronn, Germany). Thirty microliters of a 10^−7^ M HRP solution were added into a 3 mL quartz cuvette (pathlength of 1 cm, Agilent, USA) containing 2.96 mL of a 0.3 mM ABTS solution in phosphate-citrate buffer (51 mM Na_2_HPO_4_, 24 mM citric acid, pH 5.0) and stirred. In this way, the final HRP concentration in the cuvette was 10^−9^ M. Finally, eight-point-five microliters of 3% (*w*/*w*) H_2_O_2_ were added into the cuvette. Spectrum acquisition was started immediately upon the addition of H_2_O_2_.

## 3. Results

### 3.1. Atomic Force Microscopy

**Figure 2:**
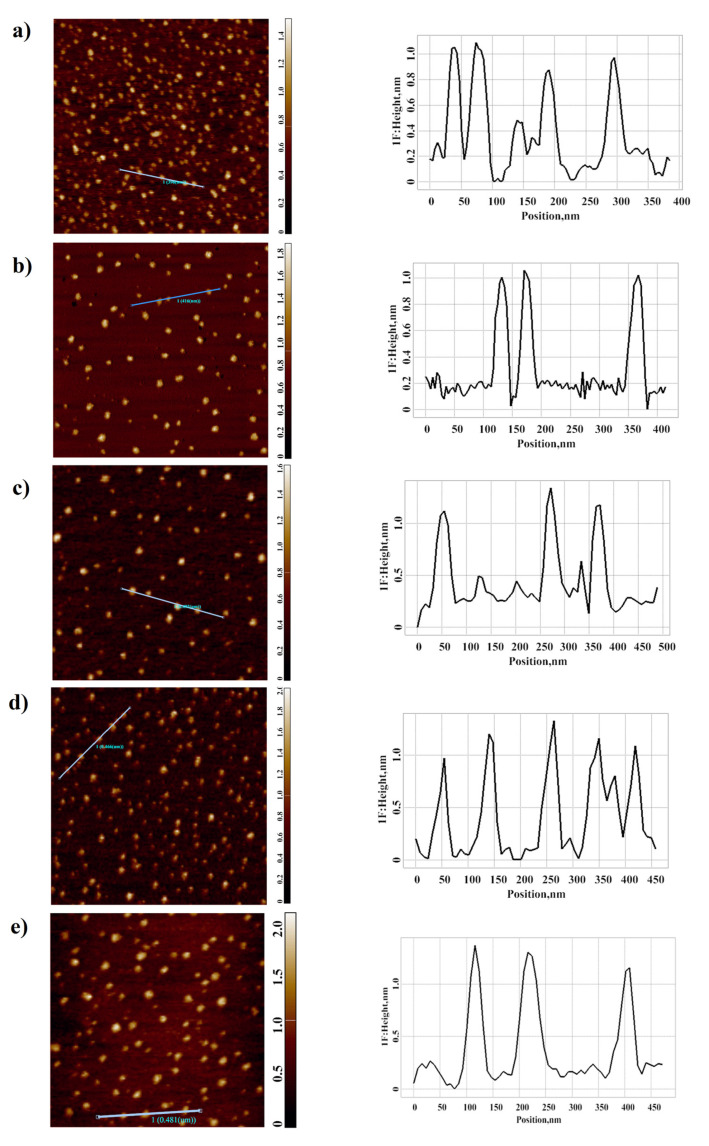
Results of AFM experiments. Typical AFM images of the surface of mica substrates with adsorbed HRP macromolecules (left) and cross-section profiles (right), corresponding to the lines in the AFM images. The analyzed HRP solution was incubated either 2 m away from the metallic half-sphere (**a**) (control solution) or within the half-sphere: in its center (**b**), near its edge (**c**), and at its bottom (**d**); or in the center of the grounded sphere (**e**). Scan size: 1 μm × 1 μm (**a**–**e**); Z scale: 1.6 μm (**a**,**c**), 2.0 μm (**b**,**d**,**e**).

In our present study, two series of experiments were conducted. In the working experiments, the HRP sample solutions were incubated within the half-sphere (in its center, near its edge, or at its bottom), as shown in Figure 1a. Moreover, additional experiments were performed in order to determine how shielding of the HRP solution from external electromagnetic fields affected the measurement results. In these experiments, the HRP solution was incubated in the center of a grounded metallic sphere, as shown in Figure 1b. In control experiments, the HRP solutions were kept 2 m away from the half-sphere. Below, we present the results obtained after AFM scanning of bare mica substrates, obtained after their incubation in the analyzed HRP solutions. Analogous measurements were performed in several different locations within the half-sphere in order to find out how the incubation of the protein solution in various locations within the same half-sphere led to changes in the protein aggregation state. Namely, the HRP solutions were incubated either near the edge of the half-sphere or at its bottom (as shown in Figure 1a). Figure 2a–e displays typical AFM images of mica-adsorbed HRP, obtained in working and control experiments.

As one can see from the images shown in Figure 2, the protein adsorbed onto mica in the form of isolated compact objects.

**Figure 3:**
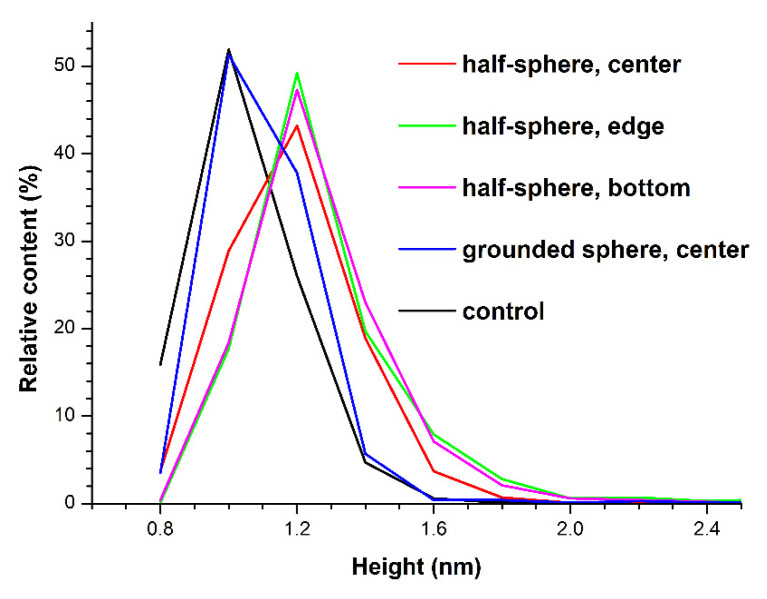
Results of AFM data processing. The plots of the density of the relative distribution of the imaged objects with height *ρ(h)* (density function plots) obtained for the HRP solutions, which were incubated either 2 m away from the metallic half-sphere (black; control solution); within the half-sphere: in its center (red), near its edge (green), and at its bottom (magenta); or within the grounded metallic sphere (blue). Relative content indicates the number of protein molecules measured at a given height divided by the total number of the molecules visualized on the substrate.

After processing the AFM data, obtained for the HRP solutions, which were incubated either within the metallic half-sphere, 2 m away from it, or in the center of the grounded sphere, the corresponding density functions *ρ(h)* were plotted. Figure 3 displays the *ρ(h)* curves obtained.

The *ρ(h)* curves shown in Figure 3 indicate that in the case of the control sample, the maximum of the density function corresponded to the height of 1.0 ± 0.1 nm, while the distribution width at half-height amounted to 0.4 nm. A similar situation was observed after the incubation of the HRP solution in the center of the grounded sphere, when the maximum of the height distribution of mica-adsorbed objects was at the same position, and there was no significant change in the right wing of the height distribution at heights greater than 1.4 nm—relative to the distribution obtained for the control solution. As we justified in our previous work [4,5,6,7,10], the objects with similar heights can be attributed to the monomeric form of HRP, since the majority (~94%) of the visualized particles was no greater than 1.2 nm—which corresponds to the height of proteins of similar molecular weight [4,5,6,7,10]. At the same time, in the case of the samples incubated in the center of the ungrounded half-sphere, near its edge, or at its bottom, the maximum of the density function significantly shifted to the right, making up 1.2 ± 0.1 nm. In the case of the sample incubated in the center of the half-sphere, the distribution width at half-height increased to 0.6 nm (the same as the case with the grounded sphere), while remaining unchanged (amounting to 0.4 nm) for the samples incubated either near the edge or at the bottom of the half-sphere. The relative content of objects with a height from 1.4–3.0 nm increased from ~6% in the case of the control sample and the sample incubated inside the grounded sphere, to ~24% in the case of the sample incubated in the center of the half-sphere, and even to ~30% in the case of the sample incubated near the edge or at the bottom of the half-sphere. That is, in the protein solutions, incubated within the half-sphere—in its center, near its edge, or at its bottom—objects with greater heights appeared. Namely, aggregates with heights greater than 1.2 nm (up to 1.7 nm) were adsorbed onto mica from these solutions. That is, in the HRP solution incubated within the half-sphere, the content of objects with a greater height (which can be attributed to the aggregated form of HRP) increased.

### 3.2. Attenuated Total Reflection Fourier Transform Infrared Spectroscopy

**Figure 4:**
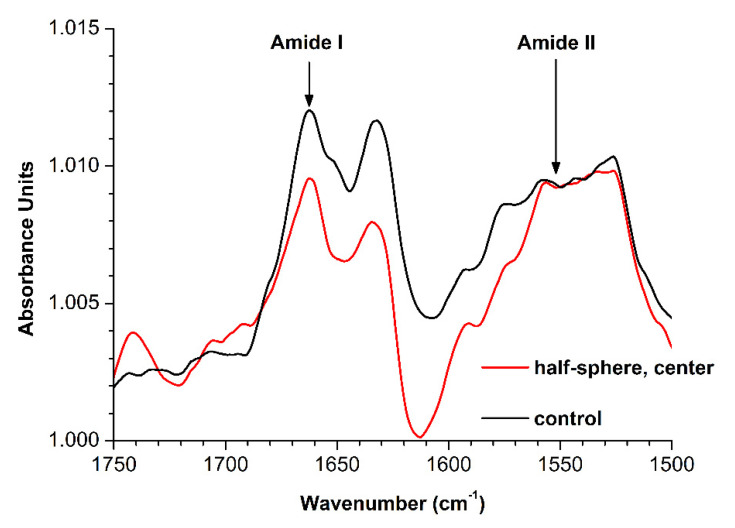
Typical attenuated total reflection Fourier transform infrared spectroscopy (ATR-FTIR) spectra of the HRP solution, incubated either in the center of the half-sphere (red) or 2 m away from the half-sphere (black). The HRP concentration was 10^−4^ M. Arrows indicate the characteristic Amide I (1660 cm^−1^) and Amide II (1550 cm^−1^) peaks.

Figure 4 displays the ATR-FTIR spectrum of the 10^−4^ M HRP solution incubated 2 m away from the half-sphere (control solution), where no influence from the half-sphere was expected.

As can be seen in Figure 4, two characteristic peaks at 1660 cm^−1^ (Amide I) and 1550 cm^−1^ (Amide II) [41] were observed for the control HRP solution, incubated 2 m away from the half-sphere, within the wavenumber range from 1500–1700 cm^−1^. ATR-FTIR measurements were also performed for the HRP solution incubated in the center of the half-sphere. The changes in this spectrum were not very strongly pronounced with respect to that of the control solution. Namely, a more considerable change in the intensity near 1660 cm^−1^ in comparison with the remaining wavenumber range studied was observed. That is, the ratio between the intensities of the Amide I and Amide II peaks changed. A slight tendency for the decrease of the intensity near 1660 cm^−1^ was observed for the spectrum of the protein solution incubated in the half-sphere in comparison with that of the control solution.

Thus, with the example of the HRP enzyme protein, the incubation of a protein solution in the center of a metallic half-sphere was demonstrated to influence its spectral characteristics.

### 3.3. Spectrophotometry-Based Estimation of HRP Enzymatic Activity

**Figure 5:**
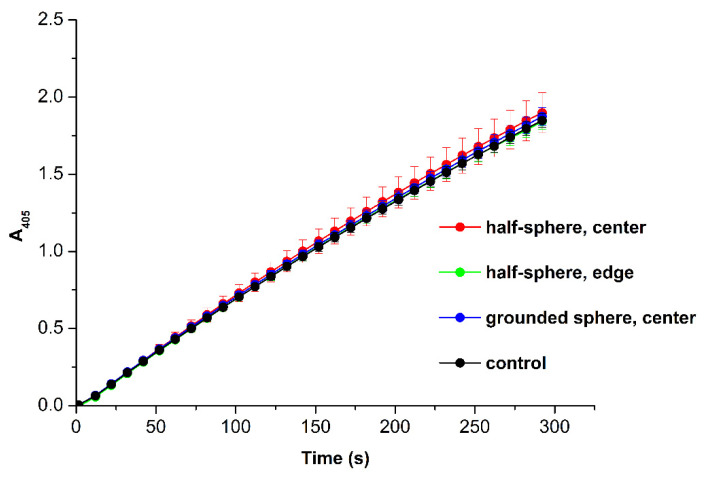
Results of spectrophotometry-based estimation of HRP enzymatic activity using a standard assay with ABTS. Typical time dependencies of the absorbance at 405 nm obtained for the HRP solutions incubated in the center of the half-sphere (red), near the edge of the half-sphere (green), in the center of the grounded sphere (green), and 2 m away from the half-sphere (control solution; black). Experimental conditions: HRP:ABTS:H_2_O_2_ = 10^−9^ M:0.3 mM:2.5 mM. T = 23 °C.

The enzymatic activity of HRP in the solutions, incubated in the center of the half-sphere, near its edge, in the center of the grounded sphere, and 2 m away from the half-sphere (control solution) was estimated by spectrophotometry as described in the Materials and Methods. Figure 5 displays the typical time dependencies of the light absorbance at 405 nm, obtained in the spectrophotometry experiments.

The time dependencies of the light absorbance at 405 nm, obtained for the HRP solutions tested, indicated no change in the enzymatic activity of the protein in our experiments. This means that the structural changes, appearing in the protein globule during the incubation of the HRP solution within the half-sphere (revealed by AFM and ATR-FTIR), did not affect its active site significantly. This is why the kinetics of the enzymatic reaction with ABTS was not affected. In other words, the functionality of the enzyme remained unchanged despite the alterations in the structure of its globule.

## 4. Discussion

Herein, the influence of a metallic half-sphere on the properties of HRP protein was studied. By AFM, an increased degree of aggregation of HRP upon its adsorption onto mica was revealed after the incubation of its solution in the center of a metallic half-sphere. The slight tendency to an alteration in the HRP secondary structure can also be indicated by comparison of the ATR-FTIR spectrum of the protein solution, incubated in the center of the half-sphere, with that of the control protein solution. At the same time, spectrophotometry measurements indicated no change in the protein’s enzymatic activity. Such a change in HRP aggregation state can be induced by alterations in the spatial structure of the protein globule. These alterations of the HRP globule’s structure, confirmed by ATR-FTIR, however, did not affect the active site, and this was why the enzymatic activity of the protein remained unchanged according to the results obtained by spectrophotometry. It is interesting to point out that an increased aggregation of mica-adsorbed HRP was also observed in the AFM experiments with the protein solutions incubated within the half-sphere (in its center, near its edge, or at its bottom). At the same time, in the additional experiments with the HRP solution, incubated in the center of the ground-shielded metallic sphere, no effect of external electromagnetic fields on both the position of the maximum and the shape of the right wing of the height distribution (at heights greater than 0.4 nm) was observed.

The protein globule structure can be affected by external background electromagnetic fields (generated by laboratory equipment), concentrated within the half-sphere at the expense of the resonance effect [16]. Namely, background electromagnetic radiation is concentrated upon its reflection from the interior surface of the half-sphere, leading to a change in the spatial topology of this radiation. Electromagnetic fields are known to influence proteins [1,3,4,5,6,7,8,9,10]. Previously, we demonstrated that even ultra-weak electromagnetic fields, whose intensity is comparable to that of the background (of 10^−12^ W/cm^2^ power density) electromagnetic fields of a non-standard specific topology—such as knotted electromagnetic fields—can induce substantial alterations in protein adsorption properties [4]. It is interesting to note that weak electromagnetic fields were shown to affect not only proteins, but also low-molecular-weight organic compounds—such as phenolic bio-antioxidants—in aqueous solutions at ultra-low concentrations (from 10^−18^–10^−15^ M). These phenomena were expected to be connected with paradoxical changes in the electrical conductivity of the solutions [42]. These paradoxical effects are discussed in the literature and are associated with quantum effects, taking into account the differences in the ortho-para isomers of water [43,44]. At the same time, it should be emphasized that the structure of HRP is rather stable, and even a 30 min exposure to a 2450 MHz microwave electromagnetic field of a standard transverse topology at 60 W radiation power does not lead to a complete loss of its activity, despite considerable alterations in its structure [9]. Notably, the HRP macromolecule is comprised of 18–27% carbohydrate chains, which stabilize the structure of the enzyme [20,22,45]. This fact can explain the high structural stability of HRP.

Thus, we demonstrated that the incubation of the protein solution in the metallic half-sphere led to a change in the aggregation state of its macromolecules, adsorbed from this solution onto mica. In our experiments, we used a half-sphere with a diameter of 30 cm. At the same time, the size of similar structures, employed in various devices, can vary over a broad range. Therefore, large spherical and half-spherical elements are widely used in bioreactors [14,15]. At the same time, spherical objects of a very small size are used in various biosensors [46]. It is known that an electromagnetic field can concentrate near the surface of metallic particles [16], and this phenomenon can be used in the development of novel highly sensitive biosensors. The development of such biosensors will help to better understand the influence of external electromagnetic fields on humans. Moreover, the development of such biosensors is quite important for the early diagnosis of somatic and infectious diseases (such as cancer, cardiovascular diseases, hepatitis, and other viral infections) in humans. For these considerations, the effect reported herein should be taken into consideration in the development of bioreactors and biosensors containing spherical elements of large or small sizes, intended for operation with protein biopolymers. The data on the effect of spherical elements on the properties of protein biopolymers are important to be taken into consideration upon the estimation of the impact of external factors on the human body in connection with the possible increased aggregation of proteins on artificial materials of a spherical shape, used in biocompatible devices implanted in the body.

## 5. Conclusions

AFM was employed to study the effect of the incubation of a protein solution in a metallic half-sphere. An increased aggregation of HRP was observed after the 40 min incubation of its 0.1 µM solution within a 300 mm titanium half-sphere, in comparison with the solution incubated inside a grounded metallic sphere and the control solution incubated 2 m away from the half-sphere. The effect observed can be explained by the influence of external background electromagnetic fields, concentrated within the half-sphere at the expense of a resonance phenomenon. These results indicated the importance of further studying how electromagnetic fields generated by various devices in both industry and everyday life can affect living systems. This is required in order to develop safety standards regulating the application of electromagnetic field-inducing equipment in industry, where technological vessels with spherical construction elements are commonly employed. The effect observed in our experiments should also be taken into consideration in the optimization of the construction of bioreactors and biosensors, intended for operation with proteins, including enzymes. This is very important upon employing enzymes, whose structure is more labile than that of HRP. Moreover, the effects of the shape of biosensors’/bioreactors’ elements on proteins are important to be taken into consideration in the development of models describing hemodynamics in the body exposed near spherical (and not only pyramidal, as discussed in the Introduction) elements for a long time, since such an exposition can cause blood supply pathologies, including thrombosis, and other systemic diseases—such as cancer and other socially significant diseases. Accordingly, the discovered effect should also be taken into consideration in the development of novel highly sensitive biosensors, intended for the early revelation of cancer and other socially significant diseases in humans.

## Data Availability

Correspondence and requests for materials should be addressed to Y.D.I.
